# Implementing quality indicators in intensive care units: exploring barriers to and facilitators of behaviour change

**DOI:** 10.1186/1748-5908-5-52

**Published:** 2010-07-01

**Authors:** Maartje LG de Vos, Sabine N van der Veer, Wilco C Graafmans, Nicolette F de Keizer, Kitty J Jager, Gert P Westert, Peter HJ van der Voort

**Affiliations:** 1 Scientific Centre for Transformation in Care and Welfare (Tranzo), University of Tilburg, PO Box 90153, Tilburg 5000 LE, the Netherlands; 2 Centre for Prevention and Health Services Research, National Institute for Public Health and the Environment, PO Box 1, Bilthoven 3720 BA, the Netherlands; 3 Department of Medical Informatics, Academic Medical Center, Meibergdreef 15, 1105 AZ, Amsterdam, the Netherlands; 4 World Alliance for Patient Safety, World Health Organization, Geneva, Schwitzerland; 5 Department of Intensive Care, Onze Lieve Vrouwe Gasthuis, Amsterdam, the Netherlands

## Abstract

**Background**

Quality indicators are increasingly used in healthcare but there are various barriers hindering their routine use. To promote the use of quality indicators, an exploration of the barriers to and facilitating factors for their implementation among healthcare professionals and managers of intensive care units (ICUs) is advocated.

**Methods**

All intensivists, ICU nurses, and managers (n = 142) working at 54 Dutch ICUs who participated in training sessions to support future implementation of quality indicators completed a questionnaire on perceived barriers and facilitators. Three types of barriers related to knowledge, attitude, and behaviour were assessed using a five-point Likert scale (1 = strongly disagree to 5 = strongly agree).

**Results**

Behaviour-related barriers such as time constraints were most prominent (Mean Score, MS = 3.21), followed by barriers related to knowledge and attitude (MS = 3.62; MS = 4.12, respectively). Type of profession, age, and type of hospital were related to knowledge and behaviour. The facilitating factor perceived as most important by intensivists was administrative support (MS = 4.3; p = 0.02); for nurses, it was education (MS = 4.0; p = 0.01), and for managers, it was receiving feedback (MS = 4.5; p = 0.001).

**Conclusions**

Our results demonstrate that healthcare professionals and managers are familiar with using quality indicators to improve care, and that they have positive attitudes towards the implementation of quality indicators. Despite these facts, it is necessary to lower the barriers related to behavioural factors. In addition, as the barriers and facilitating factors differ among professions, age groups, and settings, tailored strategies are needed to implement quality indicators in daily practice.

## Background

Quality indicators are increasingly being used in healthcare to support and guide improvements in quality of care. The purpose of implementing quality indicators as a tool to assist quality improvement is to periodically report and monitor indicator data in order to improve quality of care. In several countries, the development of indicators is emerging and examples of sets of indicators for quality of hospital care are available [1,2]. Although quality indicators are applied as a tool to guide the process of quality improvement in healthcare, hospitals that adopt quality indicators are faced with problems concerning implementation [3,4]. Successful implementation, however, is critical to maximise the effect of quality indicators on the quality of care [5].

Quality management is crucial in intensive care units (ICUs), and quality indicators can be used as a tool to assist quality improvement. Morbidity and mortality rates in ICUs vary widely among hospitals [6]. This variation is likely to be related to differences in ICU structure and care processes [7,8]. Understanding of these factors may reduce variation and ultimately improve patient care.

An evaluation of barriers to and facilitators for using quality indicators could inform strategies for their implementation in daily practice [9]. Cabana *et al. *assessed potential barriers at each stage of behavioural change for guideline implementation and placed them within a knowledge-attitude-behaviour framework [10]. Successful implementation depends upon three conditions. First, all healthcare professionals involved have to be familiar with and aware of quality indicators. Second, they need to have positive attitudes towards the use of quality indicators as a tool to improve the quality of care. Third, barriers related to behaviour, such as time and organisational constraints, need to be addressed [9,10]. Although behaviour can be changed without knowledge or attitude being affected, behaviour change based on improving knowledge and attitude is probably more sustainable than indirect manipulation of behaviour alone [10].

In general, little is known about the knowledge, attitudes, and behaviour of physicians, nurses, and managers regarding the implementation of quality indicators in daily practice. Some studies have assessed the knowledge, attitude, and behaviour of ICU staff to specific practice guidelines or guidelines in general [11-13]. Relatively few studies have examined attitudes of physicians towards the use of quality indicators; we are aware of none that have addressed the intensive care setting [14-17].

In 2006, the Dutch National Society of Intensive Care Medicine (NVIC) developed a set of quality indicators in order to evaluate and improve quality at Dutch ICUs [18]. This set of indicators is due to be implemented in all ICUs from 2008 onwards as part of the Dutch National Intensive Care Evaluation (NICE) registry. For each ICU, the implementation process of the indicators started with a course for ICU staff regarding the collection of data. This offered the possibility for the current study to explore barriers to knowledge, attitude, and behaviour that may affect implementation of quality indicators in Dutch ICUs, and to assess facilitators for the implementation of these indicators in daily practice.

## Methods

### Study population

Included in this study were all intensivists, ICU nurses, and managers (n = 142) who participated in the NICE registry course regarding the collection of indicator data [18] in the period from September 2007 to December 2008. In this study, managers were people working in the ICU who were not engaged in direct patient care but carried the responsibility of making management decisions for the ICU based on the indicator scores. Participants completed the questionnaire at the start of the training sessions.

### Questionnaire content

In close cooperation with the Dutch National Society of Intensive Care Medicine (NVIC), we developed a questionnaire that was divided into three sections.

The first part addressed professional as well as demographic characteristics such as gender, age, profession, year of graduation, and type of hospital. The second section contained statements concerning barriers at each stage of behaviour change that may affect the implementation of quality indicators in ICUs. We classified the barriers into three categories using Cabana's framework of barriers related to knowledge, attitude, and behaviour [10]. Knowledge-related barriers refer to lack of awareness or familiarity with the term quality indicator in general; barriers related to attitude refer to lack of motivation to implement and use quality indicators, or a lack of confidence in outcome. Behaviour-related barriers concern external factors such as lack of time and resources, or organisational constraints that restrict healthcare professionals' abilities to change their behaviour.

Analogous to this framework, we assessed eleven statements focusing on barriers related to knowledge, attitude, and behaviour. We used statements from a previously validated instrument designed to assess barriers to change across different innovations and healthcare settings [19]. The barriers to change in this instrument were based on a literature review and an expert panel consensus procedure with implementation experts. Studies have used this instrument successfully to identify barriers to the implementation of clinical practice guidelines [19,20]. We consulted four healthcare professionals (ICU nurses and intensivists with special interest in implementation) to check the relevance of each item on the questionnaire and whether there were any items missing that should be included. Several items in the questionnaire that were not relevant to the context of the ICU setting or to the implementation of indicators were removed. We performed an exploratory factor analysis based on current data regarding the eleven statements. This resulted in three factors, all with reasonably good reliability (Cronbach's alpha 0.73, 0.74 and 0.71) [21]. Factor one comprised two items that addressed how respondents rated their knowledge regarding quality indicators with factor loadings 0.70 and 0.81. Factor two contained six statements about their attitude with factor loadings ranging from 0.32 to 0.68, and the three statements of factor three assessed their behaviour with factor loadings 0.48, 0.50, and 0.57 respectively.

The third section of the questionnaire included questions regarding perceived facilitating factors for healthcare professionals and managers. This was based on results from a review, including studies dealing with healthcare professionals' attitude to quality and quality improvement [22]. These studies assessed healthcare professionals' enabling factors for quality improvement in healthcare.

All statements and items used in the questionnaire were scored on a five-point Likert scale ranging from '1 = strongly disagree' to '5 = strongly agree.' An open-ended question was added for additional suggestions regarding facilitating factors that might enable the implementation of quality indicators in daily practice.

### Data Analysis

Descriptive statistics were used to characterize the study sample. The questionnaire contained both positively and negatively formulated statements. To calculate a mean score, we recoded the response on the negatively formulated statements. A score of more than 3 on the five-point scale was indicated as positive, less than 3 as negative and a score of 3 was indicated as neutral. Data were excluded from analysis if there was a missing value on one or more of the items.

Multiple linear regression was used to explain the scores of the overall knowledge, attitude, and behaviour scales stratified by professional characteristics and settings. All independent variables were included into the model simultaneously, adjusting each variable in relation to the others. The scores of the overall knowledge, attitude, and behaviour scales were calculated based on the mean scores (MS) of the individual statements. The independent variables included in the model were gender, profession (healthcare professional or manager), and type of hospital (academic/teaching or non-teaching). For the purpose of analysis, respondents were divided into three age groups: <40 years, 40 to 49 years, and ≥50 years of age. In addition, MS of the reported facilitating factors among professions (intensivist, ICU nurse, and manager) were compared using analysis of variance (ANOVA) with statistical significance defined as p ≤ 0.05. The presence of multicollinearity was tested by determining the variance inflation factor (VIF) and tolerance value per variable. Cut-off values were a VIF >4 and tolerance <0.25 [23].

## Results

### Study population

All 142 professionals attending the training sessions (82 intensivists, 40 ICU nurses, and 20 managers coming from 54 ICUs in 51 hospitals out of the total of 94 Dutch ICUs) completed the questionnaire (response rate 100%). The group of participating ICUs included 36 teaching hospitals, of which six were academic hospitals (affiliated to a university), and 15 were non- teaching hospitals. The characteristics of the respondents are shown in Table 1. The majority of the 142 respondents were male (66%), 71% graduated after 1990, 50% were between 40 and 50 years of age, and 76% were affiliated to teaching or academic hospitals.

**Table 1:** Study population (n = 142)

Demographics and professional characteristic	n	**(%)**^**a**^
**Gender**		
Male	93	(65.5)
Female	49	(34.5)

**Age (years)**		
<40	42	(29.6)
40 to 49	72	(50.7)
>49	28	(19.7)

**Profession**		
Intensivist	82	(57.7)
ICU nurse	40	(28.2)
Management	20	(14.1)

**Hospital type**		
Academic hospital	12	(8.5)
Teaching hospital	96	(67.6)
Non-teaching hospital	34	(23.9)

**Year of graduation**		
1971 to 1990	41	(29.1)
>1990	100	(70.9)

^a ^Numbers may not add up to 142 and percentages may not add up to 100% due to missing values

### Barriers regarding knowledge, attitude, and behaviour

**Figure 1:**
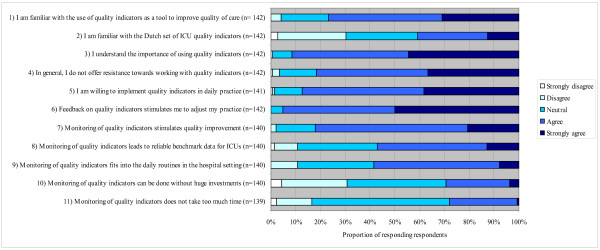
**Response to eleven statements regarding perceived barriers towards implementation of quality indicators**.

Figure 1 shows the response to each of the eleven statements. Seventy-seven percent of the respondents were familiar with the use of quality indicators as a tool to improve quality of care, and 41% knew about the Dutch set of ICU quality indicators (statements one and two).

Scores on attitudes varied between 55% of the respondents agreeing that monitoring of quality indicators leads to reliable benchmark data for ICUs, up to 95% of the respondents agreeing that receiving feedback on quality indicators stimulated them to adjust their practice (statements three through eight). More than 90% reported understanding the importance of using quality indicators and were willing to implement quality indicators in daily practice. Approximately 80% agreed with the statement that they would not resist working with indicators in the near future and agreed that monitoring of quality indicators stimulates quality improvement.

As shown in Figure 1, 59% percent of all respondents agreed that monitoring of quality indicators fits into the daily routines in the hospital setting, 28% agreed that monitoring does not take too much time, and 30% agreed that monitoring of quality indicators could be done without huge investments (statements nine through eleven).

### Facilitating factors

**Figure 2:**
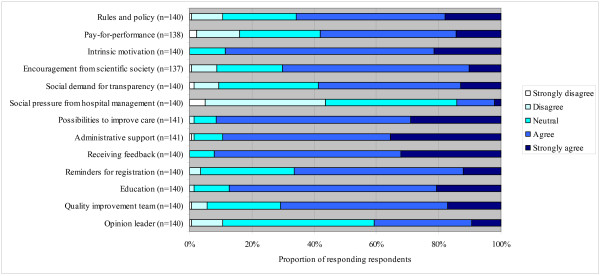
**Response to several items about factors that may facilitate the implementation of quality indicators**.

In regard to the perceived facilitating factors, respondents reported receiving feedback on quality indicator data (92% of the respondents), administrative support (89%), and education (87%) as important facilitating factors (see Figure 2). Factors related to the intrinsic motivation of healthcare professionals and managers for improvement (90%) and possibilities to improve care (91%) were also considered as important facilitators. The least perceived as facilitating factors were those related to external motivation, such as social pressure from hospital management (14%), pay for performance (57%), social demand for transparency (58%), and the designation of an opinion leader (41%).

Of the 142 respondents, 77 reported additional suggestions regarding facilitating factors for the implementation of quality indicators. Most of the responses revealed factors relating to the availability of resources such as the implementation of a patient data management system (PDMS) coupled with a hospital information system (20% of 77 respondents) and user-friendly software to register the indicators (9%). Other suggestions regarding resources are the appointment of one person responsible for the coordination and registration of the indicators (for example, a secretary) (8%), additional staff members for administrative support (10%), additional hours for non-patient related work such as the registration of the indicators (9%), the establishment of a quality improvement (QI) team (5%), support from management (4%), and the appointment of a quality manager at the ICU (3%). In addition, respondents offered some suggestions regarding education. Most respondents reported the importance of well-trained personnel for the indicator registration (14%), availability of information about the purpose and importance of using indicators (8%), and education in quality improvement principles (7%). Regarding feedback, some respondents suggested that the frequency of the feedback should be quarterly and be provided by mail (7%), and should also give alerts when one exceeded predefined targets (3%). With respect to the content of the feedback, respondents reported that feedback should be confidential, independent, and positive (4%). In addition, the content of the feedback should include results at ward level with comparison to the national benchmark and to similar units (3%). Finally, respondents state that it is important that the recipients of the feedback include ICU management and healthcare professionals (2%).

### Determinants of knowledge, attitude, and behaviour

Collinearity statistics did not show any variables with a VIF > 4 or a tolerance < 0.25. Therefore, all previously described independent variables were included in the multiple linear regressions. Table 2 illustrates the determinants of self-reported scores on overall knowledge, attitude, and behaviour determined from regression analyses. The multiple linear regression showed that being a manager (β = 0.58; p = 0.00) and being between 40 and 49 years old (β = 0.35; p = 0.03) were related to a higher level of overall knowledge. Managers had a higher level of knowledge compared to healthcare professionals (MS = 4.1 versus MS 3.5; p = 0.004). Within the group of healthcare professionals, ICU nurses had a lower level of knowledge than intensivists (MS = 3.1 versus MS = 3.7; p = 0.01).

**Table 2:** Determinants of scores on overall knowledge, attitude, and behaviour scale

	Overall knowledge*		Overall attitude*		Overall behaviour*	
	Beta	P-value	Beta	P-value	Beta	P-value
Constant	3.37	0.00	4.06	0.00	3.18	0.00
Manager (versus healthcare professional)	0.58	0.00	0.20	0.10	0.07	0.63
Female (versus male)	0.04	0.79	0.05	0.61	-0.03	0.81
Aged between 40 and 49 years (versus aged <40 years)	0.35	0.03	0.01	0.88	0.05	0.65
Aged >49 years (versus aged <40 years)	0.31	0.13	0.16	0.20	0.36	0.01
Non-teaching hospital (versus academic or teaching)	-0.32	0.05	-0.09	0.35	-0.29	0.01

Mean values and P-values were obtained by multiple linear regression (n = 142), involving all variables simultaneously.

*Overall = all statements combined

In addition, working in a non-teaching hospital was associated negatively with overall knowledge (β = -0.32; p = 0.05) (Table 2). Healthcare professionals and managers working in non-teaching hospitals had a lower level of knowledge compared to those working in academic or teaching hospitals (p = 0.01).

None of the characteristics was statistically significant related to overall attitude (Table 2). The multiple linear regression revealed that being older than 49 years (as compared to colleagues under 40 years of age) positively affected overall behaviour (β = 0.36; p = 0.01), whereas working in a non-teaching hospital was negatively associated with high scores on the overall behaviour scale (p = 0.01).

### Determinants of facilitating factors

The perceived facilitating factors differed among the various types of professions. Intensivists reported administrative support as the strongest facilitating factor (MS = 4.3; p = 0.02), ICU nurses reported education as being the most important (MS = 4.0; p = 0.01), and managers indicated receiving feedback (MS = 4.5; p = 0.001) and opportunities to improve care (MS = 4.5; p = 0.003) as the most important facilitating factors. Intensivists, nurses, and managers perceived social pressure from hospital management as the least facilitating factor (MS = 2.6; 2.8 and 2.8, respectively).

## Discussion

We conducted an exploratory study of self-reported barriers to and facilitators for the implementation of quality indicators in Dutch ICUs. Our results show that, in general, healthcare professionals and managers are familiar with the concept of using quality indicators to improve care. Although they have positive attitudes regarding the implementation of quality indicators, many are less than confident that these indicators can be fully implemented in their daily practice. These findings in the ICU setting are in line with previous results outside the ICU, which indicate that even if healthcare professionals are familiar with indicators and have overall positive attitudes regarding quality indicators, there is no guarantee that they will change their daily practice [24,25]. Lack of time and resources can be considered as the most important barriers to the implementation of quality indicators in Dutch ICUs.

The facilitating factors most frequently mentioned in this study were related to the availability of resources such as a PDMS interfaced with a hospital information system and user-friendly software to register the indicators. Other important factors were the designation of well-trained persons to carry out the indicator data registration. These results are similar to the findings of other international studies [20,22].

Our results show that respondents' profession, age, and type of hospital were associated with certain aspects of knowledge and behaviour. Familiarity with the use of quality indicators as a tool to improve the quality of care was higher among intensivists and managers, compared to nurses. Nurses were also less familiar with the Dutch set of ICU quality indicators. In order to become more familiar with the set of ICU quality indicators, it may be necessary to provide them with additional training, including handbooks and instructions on how to collect data. Healthcare professionals and managers between 40 and 49 years old and working in academic or teaching hospitals had a higher overall knowledge level, compared to those younger than 40 and those working in non- teaching hospitals. This finding is consistent with a recently conducted study that reported that older healthcare professionals working in the ICU had more knowledge of guidelines compared to younger healthcare workers [12]. None of the characteristics included in our analyses was a significant predictor of overall attitude. Regarding behaviour-related barriers, higher age and working at academic or teaching hospitals were significant predictors. Healthcare professionals and managers working at ICUs in academic and teaching hospitals tend to be more prepared to change behaviour and to actively work towards implementation compared to healthcare professionals and managers working in ICUs in non-teaching hospitals.

In our sample, non-teaching hospitals are slightly underrepresented compared to the overall proportion of non-teaching hospitals nationwide in the Netherlands. This may indicate that the results are somewhat more positive, because these hospitals showed lower scores on knowledge and behaviour in our study. However, generalisability to all Dutch ICUs is not the main objective of the current study. The study aims to identify barriers as perceived by healthcare professionals who already work with indicators. In addition, the proportion of non-teaching hospitals in our study is similar to the proportion of non-teaching hospitals participating in the Dutch NICE registry, which may indicate that non-teaching hospitals are less motivated to implement quality indicators in daily practice. Haagen *et al. *[26] also found that working in a non-teaching hospital is related to barriers regarding motivation.

Several factors can be of importance in facilitating the implementation of quality indicators. Our study showed that intrinsic motivation and possibilities to improve care are considered as very important facilitating factors. Consistent with results from other studies, factors such as administrative support and receiving feedback were also considered as important facilitators [18,27]. Intensivists, nurses, and managers appear to have different ideas concerning the perceived facilitating factors. Nurses were less familiar with quality indicators and reported that they would like to have some training in the registration of the indicators. Managers prefer to receive feedback on indicator scores, and intensivists reported administrative support as the most important facilitating factor. These findings imply that in order to implement quality indicators successfully in the ICUs, different strategies for different types of professionals are needed.

This study was a first exploration of barriers to and facilitators for the implementation of quality indicators in ICUs. The sample of respondents represented healthcare professionals who volunteered to attend training sessions aiming to implement quality indicators at their ICU. Therefore, the results might give a somewhat more positive picture than is the case elsewhere because these respondents may be more motivated compared to the total population of ICU professionals. In addition, because the 54 ICUs represented in our sample represent 57% of Dutch ICUs, results may not be generalisable to all ICUs. However, it serves as a valuable first attempt to evaluate attitudes of healthcare professionals and managers towards implementation of quality indicators in daily practice. Whether these results can be extrapolated to other countries can only be a matter of speculation. However, we cannot think of obvious reasons why other developed countries would yield different results.

This study relies on self-reported perceived knowledge, attitude, and behaviour. Inevitably there is a risk of social desirability bias (individuals may wish to present themselves or their organisation in a favourable way). Nevertheless, these data provide evidence of the barriers and facilitators that exist in regard to the implementation of quality indicators in ICUs and provide useful suggestions for the implementation. Administrative support, additional education, and effective feedback of indicator scores may be effective strategies to lower the barriers. In addition, special attention needs to be paid to healthcare professionals working in ICUs in non-teaching hospitals in order to motivate them to implement quality indicators, and to the education in quality improvement concepts for both those working in ICUs in non-teaching hospitals and nurses. This difference in focus should be taken into account when developing implementation strategies. Tailored strategies have to be developed for each profession or type of hospital.

Because no validated questionnaires were available on this subject, we developed our own questionnaire. In this, we used the well-known framework of Cabana evaluating the stages of behaviour change. We reformulated the statements regarding barriers to guideline adherence because we used the classification within the framework of identifying barriers to implementing indicators in daily practice. Although the value of the questionnaire needs to be confirmed, inspection of the factor loadings and internal consistency suggests that it could be a useful tool for future studies.

## Summary

In conclusion, the results of this study suggest that even in a situation in which knowledge and attitude towards implementation are generally positive, barriers related to behaviour need to be addressed before healthcare professionals and managers would be willing to work actively towards implementation.

Despite the increased interest in using quality indicators in daily practice in order to improve the quality of care, hospitals often struggle with its implementation [3,5,28]. The present exploratory study is the first study with a structured method to identify important barriers and facilitators that might inform the process of implementation. It could serve as a starting point for professionals and organisations to identify local barriers in more detail and to develop tailored strategies for the implementation of quality indicators in their organisation. Moreover, we have used these findings as a part of the development of a tailored strategy to address these barriers in order to improve the implementation of quality indicators in clinical practice.

## Competing interests

The authors declare that they have no competing interests.

## Authors' contributions

All authors participated in manuscript preparation, and read and approved the final manuscript.
